# Assessing psychometric properties of the revised Richards-Campbell Sleep Questionnaire based on COSMIN: a systematic review

**DOI:** 10.3389/fmed.2026.1826844

**Published:** 2026-05-07

**Authors:** Yanting Zhang, Jing Ming, Chong Cheng, Yuan Zhang, Yihua Yang, Xinbo Ding, Jing Ma

**Affiliations:** Department of Critical Care Medicine, Hubei Clinical Research Center for Critical Care Medicine, Zhongnan Hospital of Wuhan University, Wuhan, China

**Keywords:** attribute of measurement, COSMIN checklist, RCSQ, Richards-Campbell sleep scale, systematic review

## Abstract

**Objective:**

The study aimed to systematically review the measurement properties of the revised Richards–Campbell Sleep Questionnaire (RCSQ) and the methodological quality of the studies to provide evidence for research related to sleep assessment in intensive care unit (ICU) patients.

**Methods:**

We searched PubMed, Embase, CINAHL, Web of Science, CNKI, Wanfang, VIP, and SinoMed from inception to 10 August 2024. This study was conducted in accordance with the PRISMA 2020 guideline. We used the COnsensus-based Standards for the selection of health Measurement INstruments (COSMIN) Risk of Bias (RoB) checklist and the JBI cross-sectional study evaluation criteria to evaluate the methodological quality of the included studies, and the COSMIN quality criteria to assess the measurement properties of the revised RCSQ.

**Results:**

A total of 14 studies were included, covering eight language versions of the revised RCSQ, mostly from Asian countries. The samples included ICU patients, older inpatients, orthopedic perioperative patients, and acute stroke patients. Internal consistency was good across most versions, but content validity, structural validity, cross-cultural validity, and measurement error were underreported or insufficient. Methodological quality showed some risk of bias, and the evidence across versions was inconsistent.

**Conclusion:**

The revised RCSQ versions show good internal consistency but lack robust validation for key measurement properties. Future studies should follow updated COSMIN guidelines to improve design, reporting, and quality of evidence.

**Systematic review registration:**

PROSPERO registration number CRD42023452082.

## 1 Introduction

Sleep is particularly important for the recovery of both psychological and physiological functions. However, patients in the intensive care unit (ICU) are susceptible to severe sleep disturbances due to factors such as mechanical ventilation, sedative medications, continuous noise and light, and frequent repositioning for physical examination (1). Sleep disorders are widespread and have a serious impact on patients’ physiology, psychology, and prognosis, yet this issue is often overlooked (1, 2). Compared to healthy adults, studies using polysomnography (PSG) to investigate sleep disorders in ICU patients have shown that sleep deprivation is characterized by prolonged sleep latency, sleep fragmentation, decreased sleep efficiency, multiple awakenings, predominance of stage 2 sleep, reduced or absent stage 3 sleep (deep sleep), and reduced or absent rapid eye movement (REM) sleep (3–6). Although the average total sleep time is not significantly different from that of healthy adults, approximately 50% of ICU patients’ sleep occurs during the daytime, with a significant shift toward lighter sleep stages (7, 8). These findings have been consistently observed across different ICU settings (7, 8).

Sleep deprivation in the ICU has negative effects on patient treatment outcomes, such as impaired wound healing, reduced anti-infection capacity, and decreased treatment compliance. It may even lead to delirium, prolong recovery time and hospital stay, and, in severe cases, result in ICU syndrome characterized by agitation, emotional irritability, and confusion, thereby increasing morbidity and mortality (9). Therefore, quantitative sleep assessment, screening for poor sleep quality, and targeted interventions are clinically important to reduce delirium rates, shorten ICU length of stay, and improve patient recovery (10).

Currently, sleep assessment methods are divided into objective and subjective methods. Objective assessment includes polysomnography (PSG), the “gold standard” for sleep measurement, as well as the bispectral index, actigraphy, and endogenous melatonin measurement. Melatonin measurement is primarily used to assess peripheral circadian rhythms; although it provides objective data related to sleep–wake cycles, it is not a direct measure of sleep itself but rather an indicator of circadian alignment. However, these objective sleep assessment methods are difficult to implement widely in the ICU due to high equipment and material costs, as well as high technical requirements. Subjective sleep assessment includes the nurse observation method, which is easy to implement in nursing practice, but research (11) has shown that nurses tend to overestimate the quality and quantity of patients’ sleep, resulting in poor reliability of the results, making this method unsuitable for ICU patients. Another approach is the questionnaire method, including patient self-assessment questionnaires and proxy evaluation, which is more economical, time-saving, and labor-efficient; however, it lacks standardization. Most subjective assessment scales contain numerous items and complex wording, making them unsuitable for use in ICU patients (12). Therefore, it is necessary to select a simple and reliable sleep assessment tool to evaluate the sleep status of ICU patients, thereby quantifying sleep and assessing the effectiveness of sleep intervention strategies.

American scholars Richards et al. (13) designed the Richards–Campbell Sleep Questionnaire (RCSQ) specifically for sleep assessment in ICU patients. With its simple structure and ease of use for patients, it is considered a potential sleep assessment tool that can be widely applied in ICU settings. It has been translated into different language versions, and its performance has been evaluated, making it the most widely used tool for clinical sleep quality assessment in ICU patients (14–16). However, good psychometric properties are a fundamental prerequisite for the effective use of a scale. To date, there has been no systematic evaluation of the RCSQ’s measurement properties, which may introduce bias and affect the reliability of sleep assessment results. Various methods are available for evaluating the measurement properties of patient-reported outcome measures (PROMs), among which the COnsensus-based Standards for the selection of health Measurement INstruments (COSMIN) is a widely recognized framework (17). It can assess multiple indicators of scale quality, including reliability, validity, and responsiveness. Meanwhile, through comprehensive quality evaluation and assessment of a scale’s measurement properties, COSMIN promotes the scientific and standardized development of health outcome measurement tools.

The revised RCSQ retains the same five core dimensions and item structure as the original version; only linguistic and cultural adaptations were made, without changing core items or scoring rules. This systematic review aimed to evaluate the measurement properties of revised RCSQ versions as patient-reported outcome measures (PROMs) for hospitalized and critically ill patients, following COSMIN methodology.

## 2 Study design

This systematic review was performed in accordance with PRISMA 2020, the JBI guidelines for systematic reviews of measurement properties (18), and COSMIN methodology for systematic reviews of outcome measurement instruments. We followed the Meta-analyses of Observational Studies in Epidemiology (MOOSE) guidelines for reporting (19).

## 3 Study methods

### 3.1 Literature search

We searched eight databases from inception to 10 August 2024: PubMed, Embase, CINAHL, Web of Science, CNKI, Wanfang, VIP, and SinoMed. Search terms included the following: (“Richards-Campbell Sleep Questionnaire” OR “RCSQ”) AND (“sleep” OR “sleep quality” OR “sleep disturbance”) AND (“intensive care” OR “ICU”) AND (“reliability” OR “validity” OR “psychometric” OR “measurement properties” OR “cross-cultural adaptation”). We did not include grey literature or trial registries.

English search terms: For PubMed, Step 1: (Richards-Campbell Sleep Questionnaire) OR (RCSQ); Step 2: (sleep OR “sleep quality” OR “sleep disturbance” OR “sleep assessment”) AND (“intensive care unit” OR “ICU” OR “critical care”); Step 3: We used the highly sensitive search strategy developed by Terwee et al. (20) to identify studies on the psychometric properties of scales. Search strategies and results for other databases are shown in the annex. The detailed search strategies are presented in Supplementary File 1.

The literature search was conducted from the inception of each database to 10 August 2024. We did not update the search after this date because a supplementary search was performed in February 2026 (before manuscript submission), and no new studies meeting the inclusion criteria (i.e., studies reporting the psychometric properties of the revised RCSQ) were identified. Thus, the original search results are comprehensive and cover all relevant studies to date.

### 3.2 Literature inclusion and exclusion criteria

The inclusion criteria were as follows: Population: Hospitalized patients; content: Studies reporting the measurement properties of revised RCSQ versions; language: Chinese or English; and study design: Cross-sectional, cohort, or descriptive studies reporting psychometric data.

The exclusion criteria were as follows: Non-revised RCSQ or non-target populations; studies using the RCSQ only as an outcome without psychometric evaluation; reviews, meta-analyses, case reports, letters, and conference abstracts; unavailable full texts; duplicate publications; and studies published in languages other than Chinese or English.

The rationale for excluding non-psychometric studies was as follows: Clinical trials rarely report the complete data required for COSMIN evaluation (e.g., structural validity and measurement error), and including them would reduce comparability and compromise the risk of bias assessment. This is a limitation but is consistent with the aim of the review.

Randomized controlled trials (RCTs) were excluded from this review because most RCTs only use the RCSQ as an outcome measure to evaluate the effect of interventions and do not report the complete psychometric data (e.g., structural validity and measurement error) required for COSMIN evaluation. Including RCTs would reduce the comparability of the study results and the accuracy of the bias risk assessment, which is inconsistent with the objective of this systematic review.

### 3.3 Literature screening and information extraction

Literature screening and data extraction were independently conducted by two researchers trained in evidence-based practice and proficient in the JBI cross-sectional study evaluation criteria (21) and the COSMIN health measurement tool selection criteria (19). In case of disagreement, a third researcher was consulted to reach a consensus. The researchers first conducted an initial screening based on the title and abstract, excluding studies that clearly did not meet the inclusion criteria. Then, they read the full text of the remaining literature for further screening to make the final inclusion decision. The extracted data included the first author, publication year of the revised version, country, study design, participant characteristics (source, sample size, age, and sex distribution), and relevant information on the revised RCSQ (e.g., Cronbach’s *α* coefficient, test–retest reliability, content validity, construct validity, and criterion validity). In this study, the agreement rate for literature screening was 93.5% (*κ* = 0.87), and the agreement rate for quality assessment was 91.2% (κ = 0.83), indicating good inter-rater agreement between the two researchers.

### 3.4 Quality evaluation

#### 3.4.1 Quality evaluation of the literature

The JBI cross-sectional study evaluation criteria are widely used for assessing the methodological quality of cross-sectional studies and include eight key evaluation items (e.g., clear definition of sample inclusion criteria, detailed description of study participants and settings, and valid and reliable measurement of exposure and outcomes). Application method: Two independent researchers rated each included study against each item using four response options: “Yes,” “No,” “Unclear,” or “Not applicable.” Result interpretation: Studies with a “Yes” rate >70% were defined as high quality, those with 40% ~ 70% as moderate quality, and those with <40% as low quality.

#### 3.4.2 Quality evaluation of the revised scale

##### 3.4.2.1 Methodological quality assessment

The studies included in this systematic review did not involve scale development, and since the content structure of the scale has not changed, the COSMIN risk of bias (RoB) assessment checklist (22) was used to evaluate the methodological quality of the revised scale, covering eight measurement properties: Construct validity, internal consistency, cross-cultural validity/measurement invariance, stability, measurement error, criterion validity, hypothesis testing, and responsiveness. A 4-point Likert scale was used for scoring: “very good,” “good,” “doubtful,” and “inadequate.” The minimum scoring principle was adopted to determine the overall methodological quality of each measurement property; that is, the overall risk of bias score for an attribute is determined by the lowest score among all items within that attribute. If some items have a “not applicable” option, they are not considered in this principle (22).

##### 3.4.2.2 Evaluation of measurement characteristics

The COSMIN quality standards (17) were used to evaluate the eight measurement properties of the questionnaire, which were classified into three categories: “sufficient (+),” “insufficient (−),” and “uncertain (?).” If the evaluation results for a measurement property across the included studies were consistent (all sufficient, all insufficient, or all uncertain), the overall rating for that measurement property was the corresponding grade. If the evaluation results for a measurement property were inconsistent across studies and the reason for the inconsistency was not explained, the overall rating for that measurement property was classified as “inconsistent (±).”

##### 3.4.2.3 Definitions of key measurement properties

Based on the 2018 COSMIN guideline, the key measurement properties of the revised RCSQ evaluated in this review are defined as follows: Structural validity: The degree to which the scale scores reflect the proposed theoretical construct of sleep quality in critically ill patients, as evaluated by factor analysis. Internal consistency: The degree of intercorrelation among the scale items, reflecting the homogeneity of the measurement construct, measured using Cronbach’s *α* coefficient. Cross-cultural validity/measurement invariance: The degree to which the scale’s measurement construct and item performance are consistent across different cultural and linguistic groups. Stability (test–retest reliability): The consistency of the scale scores obtained from the same respondents at different time points, measured using the intraclass correlation coefficient (ICC). Measurement error: The random error in the scale scores, reflecting the precision of the measurement. Criterion validity: The degree of agreement between the scale scores and a well-validated gold standard that directly measures the same construct. Hypothesis testing: The verification of *a priori* hypotheses about the relationship between the scale scores and other relevant measures, reflecting the theoretical consistency of the construct. Responsiveness: The ability of the scale to detect clinically important changes in sleep quality over time in critically ill patients.

#### 3.4.3 GRADE-adapted certainty of evidence assessment

According to the 2018 COSMIN guideline, we conducted a GRADE-adapted certainty of evidence assessment for each measurement property of the revised RCSQ and each language version. The certainty of evidence was rated on a four-level scale: High, moderate, low, and very low. The ratings were based on five key factors: Risk of bias in the included studies, inconsistency of the results, indirectness of evidence, imprecision of the data, and publication bias. A total of two independent researchers completed the ratings, and discrepancies were resolved by a third researcher.

### 3.5 Statistical methods

In this study, descriptive analysis was used to summarize the basic characteristics of all included studies and present them in tables. A meta-analysis was not performed for the following reasons: First, the included studies varied in terms of sample size, population characteristics (e.g., different disease types and age groups), and evaluation methods of measurement properties, leading to significant heterogeneity that could not be effectively adjusted. Second, the reporting of measurement properties across studies was inconsistent (e.g., some studies only reported internal consistency, while others reported limited validity data), making it difficult to conduct a quantitative synthesis of the results. Therefore, a narrative synthesis of the data was deemed more appropriate to comprehensively present the findings.

## 4 Results

### 4.1 Study selection

**Figure 1:**
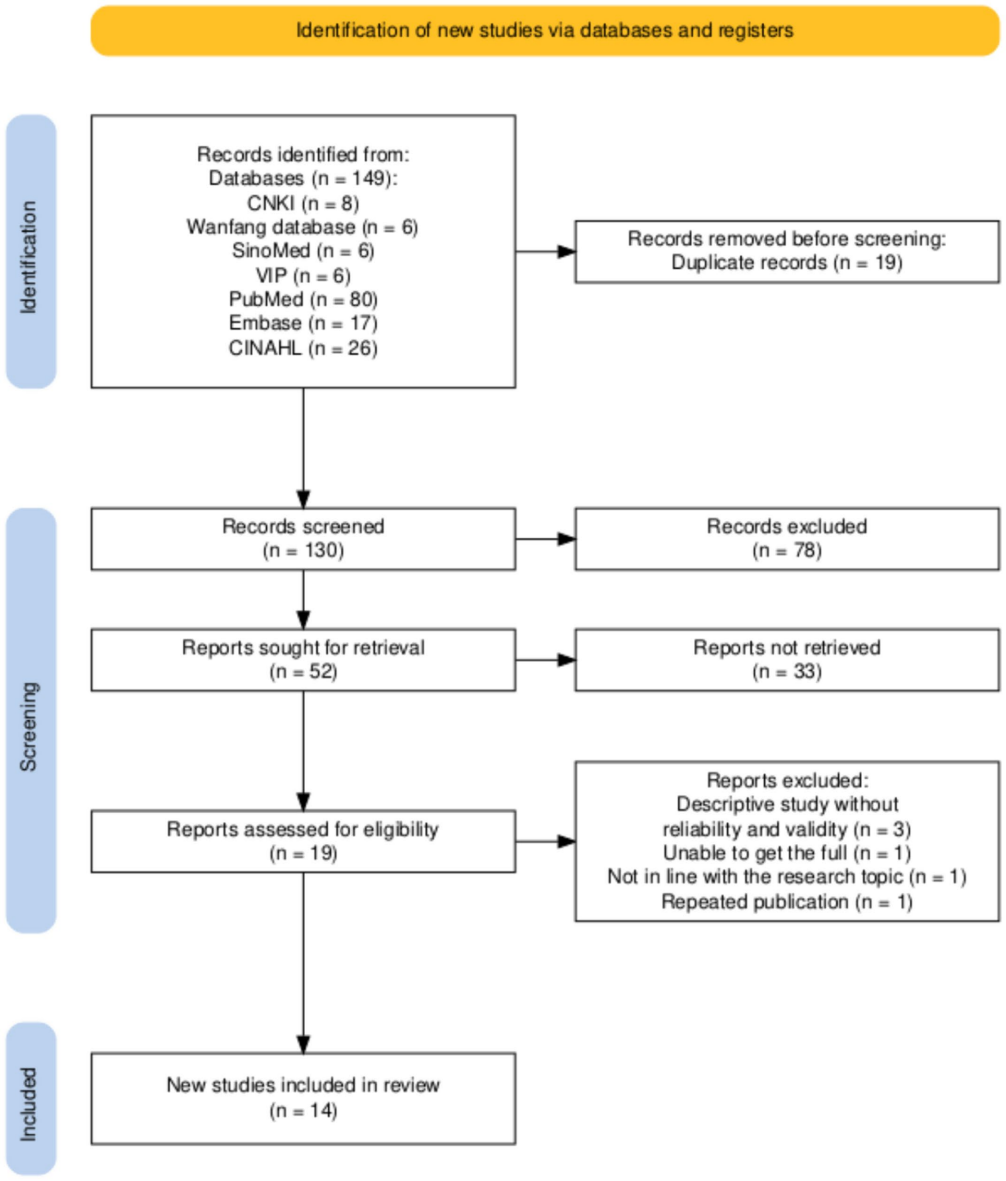
PRISMA 2020 flow diagram of the literature screening and study selection process.

A total of 147 relevant studies were initially identified through systematic searches of the eight databases. After removing 19 duplicates, 128 studies remained. Following the initial screening of titles and abstracts, 111 studies were excluded for the following reasons: 83 were irrelevant to the research theme (e.g., studies on sleep interventions that did not involve RCSQ measurement properties or studies on other sleep scales not related to the RCSQ), 26 were of excluded study types (e.g., reviews, meta-analyses, and case reports), one was published in a language other than Chinese or English, one was an informal publication (conference abstract), and two could not be retrieved. Subsequently, 19 studies were retained for full-text reading and further screening. During this stage, five studies were excluded for the following reasons: Three were descriptive studies that did not report reliability and validity data, one could not be obtained in full text, one was inconsistent with the research theme (using the original RCSQ without revision or measurement property evaluation), and one was a duplicate publication. Finally, 14 studies were included in the systematic review. The screening process is shown in Figure 1. For the specific details of the PRISMA checklist (23), please refer to Supplementary File 2.

### 4.2 Basic characteristics of the included studies

A total of 14 studies (*n* = 1,513; age 18–80 years; 53.14% male) were included. There were eight language versions (Chinese, Thai, Arabic, Portuguese, Japanese, German, Korean, and Italian). Most studies focused on ICU patients, while others included older, orthopedic, or acute stroke patients. A total of 13 studies reported Cronbach’s *α* (0.850–0.965), while six reported ICCs (0.671–0.970). In total, five studies reported criterion validity. Full details are presented in Table 1.

**Table 1:** The basic characteristics of the included studies (*n* = 14).

Studies	Year of publication (Year)	Language(Country)	Participants	Sample Size	Degree of reliability		Validity		Criterion validity	Questionnaire completion time
					Cronbach’s α	ICC	Average content validity (S-CVI/Ave)	Construct validity KMO		
Chen (30)	2016	Chinese (China)	ICU patients	127	0.895	—	0.840	0.815	0.504 ~ 0.856(SMH)	2-3 min
Yang (37)	2017	Chinese (China)	ICU patients	60	0.874	0.912	0.840	—	—	1–2 min
Huang (38)	2018	Chinese (China)	ICU patients	338	0.95	0.77	0.93	0.90	—	5-10mn
Chen (31)	2019	Chinese (China)	Older in patients	155	0.898	0.671	—	0.875	—	3 min
He (33)	2021	Chinese (China)	Orthopedic perioperative patients	199	0.965	—	0.833	0.861 ~ 0.905	−0.546 ~ −0.652(PSQI)	1-3 min
Nuanprae Kitisin (39)	2021	Thai (Thailand)	ICU patients	92	0.964	0.970	0.80	—	—	Within 15 min
Al-Sulami (24)	2019	Arabic (Arab)	ICU patients	57	0.890	—	—	—	—	—
Biazim (40)	2020	Portuguese (Brazil)	ICU patients	50	—	0.840	—	—	—	Within 30 min
Murata (34)	2019	Japanese (Japan)	ICU patients	33	0.911	—	—	—	0.459 ~ 0.602(PSG)	—
Li-Xia (32)	2018	Chinese (China)	ICU patients	150	0.923	—	0.84	0.846	0.504 ~ 0.866(SMH)	2-3 min
Krotsetis (25)	2017	German (Germany)	ICU patients	51	0.88	—	—	—		2-4 min
Varella (41)	2021	Portuguese (Brazil)	ICU patients	113	0.850	0.840	—	—	—	—
Kim (26)	2020	Korean (South Korea)	ICU patients	52	0.960	—	—	—	—	—
Rollo (35)	2022	Italian (Italy)	Patients with acute stroke	36	0.917	—	—	0.686	0.7(PSG)	—

St. Mary’s Hospital Sleep Questionnaire (SMH); Pittsburgh Sleep Quality Index (PSQI); Polysomnography (PSG). The symbol ‘-’ indicates that the relevant data were NOT REPORTED in the original study; all data in this table were extracted strictly from the original studies, and no unreported data were imputed or estimated.

Notably, the sample sizes of the included studies varied significantly, ranging from 33 to 338 participants. In total, three studies had a sample size of fewer than 100 participants, which is generally considered inadequate for psychometric evaluation—especially for structural validity and confirmatory factor analysis (CFA), which require a sufficient sample size to ensure stable and reliable results. The small sample size of these studies may lead to unstable results of structural validity evaluation, representing a potential limitation of the present study’s findings.

### 4.3 Methodological quality (JBI)

The overall quality of the 14 included studies was high: 11 studies (78.6%) had a ‘Yes’ response rate of more than 70% (defined as high quality), while the remaining three studies (21.4%) had a ‘Yes’ response rate of 40–70% (defined as moderate quality). No studies were rated as low quality (‘Yes’ response rate <40%). For the eight evaluation items of the JBI cross-sectional study evaluation criteria, three studies (24–26) were rated as “unclear” regarding “whether strategies to deal with confounding factors were stated,” while the other studies were rated “yes” for all evaluation items. To improve readability, Table 2 was reformatted with the evaluation items as column headers and the assessment results (Y/U) as cell content.

**Table 2:** Quality evaluation of the included studies (*n* = 14).

Studies	(1)	(2)	(3)	(4)	(5)	(6)	(7)	(8)
Chen (30)	Y	Y	Y	Y	Y	Y	Y	Y
Yang (37)	Y	Y	Y	Y	Y	Y	Y	Y
Huang (38)	Y	Y	Y	Y	Y	Y	Y	Y
Chen (31)	Y	Y	Y	Y	Y	Y	Y	Y
He (33)	Y	Y	Y	Y	Y	Y	Y	Y
Nuanprae Kitisin (39)	Y	Y	Y	Y	Y	Y	Y	Y
Al-Sulami (24)	Y	Y	Y	Y	Y	U	Y	Y
Biazim (40)	Y	Y	Y	Y	Y	Y	Y	Y
Murata (34)	Y	Y	Y	Y	Y	Y	Y	Y
Li-Xia (32)	Y	Y	Y	Y	Y	Y	Y	Y
Krotsetis (25)	Y	Y	Y	Y	Y	U	Y	Y
Varella (41)	Y	Y	Y	Y	Y	Y	Y	Y
Kim (26)	Y	Y	Y	Y	Y	U	Y	Y
Rollo (35)	Y	Y	Y	Y	Y	Y	Y	Y

(1) Were the criteria for inclusion in the sample clearly defined? (2) Were the study participants and setting described in detail? (3) Was the exposure measured in a valid and reliable way? (4) Were objective, standard criteria used for the measurement of the condition? (5) Were confounding factors identified? (6) Were strategies to deal with confounding factors stated? (7) Were the outcomes measured in a valid and reliable way? (8) Was appropriate statistical analysis used? “Y” means “Yes”; “U” means “unclear.”

A total of three studies were rated as ‘unclear’ for Item 6 (‘Were strategies to deal with confounding factors stated?’). The main confounding factors relevant to psychometric validation studies of the RCSQ included patient age, disease severity, educational level, and the time of scale completion (e.g., morning vs. evening). Although these three studies did not clearly state strategies for addressing confounding factors, all other seven items were rated as ‘yes.’ Therefore, the ‘unclear’ rating for a single item did not affect the overall high-quality assessment of these studies.

### 4.4 COSMIN risk of bias and measurement properties

All 14 studies were evaluated using the COSMIN 10-box framework. The key findings are as follows: (1) Structural validity was mostly rated as inadequate or doubtful, as only a few studies used confirmatory factor analysis. (2) Internal consistency was generally rated as very good or adequate, with Cronbach’s *α* values ≥0.85. (3) Reliability (test–retest) varied across studies, with ICC values ranging from 0.671 to 0.970. (4) Measurement error was reported in only one study. (5) Cross-cultural validity was not reported in any study. (6) Criterion validity findings were inconsistent, with only two studies using PSG as the gold standard. (7) Responsiveness was insufficiently reported. Full ratings are shown in Tables 3, 4.

**Table 3:** Methodological quality assessment and GRADE evidence evaluation of the revised scale (*n* = 14).

Author of the revised scale	Structural validity	Internal consistency	Stability	Criterion validity	Hypothesis testing	Responsiveness
Chen (30)	Very good	Very good	Not reported	Inadequate	Good	Good
Yang (37)	Inadequate	Very good	Very good	Not reported	Doubtful	Good
Huang (38)	Good	Very good	Very good	Not reported	Doubtful	Good
Chen (31)	Very good	Very good	Good	Not reported	Doubtful	Doubtful
He (33)	Good	Very good	Not reported	Good	Good	Good
Nuanprae Kitisin (39)	Inadequate	Very good	Very good	Not reported	Doubtful	Doubtful
Al-Sulami (24)	Inadequate	Very good	Not reported	Not reported	Doubtful	Good
Biazim (40)	Inadequate	Doubtful	Inadequate	Not reported	Doubtful	Doubtful
Murata (34)	Inadequate	Very good	Not reported	Very good	Very good	Doubtful
Li-Xia (32)	Very good	Very good	Not reported	Inadequate	Good	Good
Krotsetis (25)	Inadequate	Very good	Not reported	Not reported	Doubtful	Good
Varella (41)	Inadequate	Very good	Very good	Not reported	Doubtful	Good
Kim (26)	Inadequate	Very good	Not reported	Not reported	Doubtful	Doubtful
Rollo (35)	Inadequate	Very good	Not reported	Very good	Very good	Doubtful

**Table 4:** Quality evaluation of the revised scale (*n* = 14).

Author of the revised scale	Structural validity	Internal consistency	Cross-cultural validity	Stability	Measurement error	Criterion validity	Hypothesis testing	Responsiveness
Chen (30)	+	+	?	?	?	?	+	+
Yang (37)	−	+	?	+	?	?	?	?
Huang (38)	?	+	?	+	?	?	?	?
Chen (31)	+	+	?	−	?	?	?	?
He (33)	?	+	?	?	?	−	+	+
Nuanprae Kitisin (39)	−	+	?	+	?	?	?	?
Al-Sulami (24)	−	+	?	?	?	?	?	?
Biazim (40)	−	?	?	+	?	?	?	?
Murata (34)	−	+	?	?	?	−	+	+
Li-Xia (32)	+	+	?	?	?	?	+	+
Krotsetis (25)	−	+	?	?	?	?	?	?
Varella (41)	−	+	?	+	+	?	?	?
Kim (26)	−	+	?	?	?	?	?	?
Rollo (35)	−	+	?	?	?	+	+	+

“+” means “full”; “−” means “inadequate”; “?” means “uncertain”.

Based on the COSMIN framework and the GRADE approach to evidence grading, a comprehensive evaluation of the measurement properties of the revised RCSQ showed that (Table 5) the evidence for internal consistency was sufficient and of high quality. The Cronbach’s *α* coefficients for all language versions were ≥0.85, making it the most stable and reliable measurement property of the scale. The evidence for structural validity, criterion validity, stability (test–retest reliability), and hypothesis testing was partially sufficient and of moderate quality. Specifically, structural validity was primarily assessed using exploratory factor analysis (EFA), with only the Chinese version employing confirmatory factor analysis. For criterion validity, only a small number of studies used polysomnography (PSG) as the gold standard. The evidence for content validity, measurement error, and responsiveness was severely insufficient and of low quality. Cross-cultural validity completely lacked relevant reports, and the quality of evidence was very low. Overall, only the internal consistency of the revised RCSQ was supported by high-quality evidence, while the evidence for other key psychometric properties showed varying degrees of absence or inadequacy.

**Table 5:** Summary of evidence quality across COSMIN domains.

COSMIN domain	Sufficiency of evidence	GRADE certainty	Key findings	Overall rating
Content validity	Insufficient	Low	Reported in only a few studies; no systematic evaluation	Insufficient
Structural validity	Insufficient	Moderate	Mostly exploratory factor analysis; only Chinese versions performed CFA	Insufficient
Internal consistency	Sufficient	High	Cronbach’s α ≥ 0.85 across all language versions	Sufficient
Stability (test–retest reliability)	Partially sufficient	Moderate	Reported in some versions with variable ICC values	Uncertain
Measurement error	Very insufficient	Low	Reported in only one study (SEM/MDC)	Insufficient
Criterion validity	Insufficient	Moderate	Only two studies used PSG as the gold standard	Insufficient
Hypothesis testing	Partially sufficient	Moderate	Most studies performed correlation analyses but lacked standardized methods	Uncertain
Cross-cultural validity	Completely lacking	Very low	No study reported measurement invariance	Insufficient
Responsiveness	Very insufficient	Low	Rarely reported to detect clinically important change	Insufficient

### 4.5 GRADE-adapted certainty of evidence assessment

Based on the evidence quality ratings of the 14 included studies under the GRADE framework, the overall evidence quality was rated as “moderate to high”: Seven studies (50%) were classified as high-quality evidence, while the other seven studies (50%) were classified as moderate-quality evidence, with no studies rated as low or very low quality. Across all included studies, the domains of risk of bias, inconsistency, indirectness, and publication bias were assessed as “low”; differences were only observed in imprecision. Specifically, studies rated as high-quality evidence met the criteria of adequate sample size (≥100 cases) and comprehensive validation of measurement properties and were primarily concentrated in the Chinese, Thai, and Brazilian Portuguese versions. In contrast, studies rated as moderate-quality evidence were characterized by smaller sample sizes (33–60 cases) or partial validation of measurement properties and were mainly associated with smaller-sample language versions such as the Japanese, Korean, and German versions, as well as studies with single-domain validation. Overall, these variations did not compromise the reliability of the core conclusions (Table 6).

**Table 6:** GRADE-adapted certainty of evidence assessment.

Author of the revised scale	Risk of bias	Inconsistency	Indirectness	Imprecision	Publication bias	Grade evidence level
Chen (30)	Low	Low	Low	Low	Low	High
Yang (37)	Low	Low	Low	Medium	Low	Medium
Huang (38)	Low	Low	Low	Low	Low	High
Chen (31)	Low	Low	Low	Low	Low	High
He (33)	Low	Low	Low	Low	Low	High
Nuanprae Kitisin (39)	Low	Low	Low	Low	Low	High
Al-Sulami (24)	Low	Low	Low	Medium	Low	Medium
Biazim (40)	Low	Low	Low	Medium	Low	Medium
Murata (34)	Low	Low	Low	High	Low	Medium
Li-Xia (32)	Low	Low	Low	Low	Low	High
Krotsetis (25)	Low	Low	Low	Medium	Low	Medium
Varella (41)	Low	Low	Low	Low	Low	High
Kim (26)	Low	Low	Low	Medium	Low	Medium
Rollo (35)	Low	Low	Low	High	Low	Medium

## 5 Discussion

Overall, the revised RCSQ shows consistent and good internal consistency across all language versions and study populations (Cronbach’s *α* 0.850–0.965), which is the most robust measurement property of the scale. However, the evidence for most other key measurement properties is insufficient or indeterminate: Content validity and cross-cultural validity are severely underreported, structural validity is limited by inappropriate factor analysis methods and small sample sizes, criterion validity is inconsistent due to improper gold standard selection, and measurement error and responsiveness are rarely reported. These deficiencies indicate that the psychometric validation of the revised RCSQ is still incomplete, and further high-quality validation studies are needed.

### 5.1 Content validity and structural validity

Based on the JBI critical appraisal checklist for cross-sectional studies, the overall methodological quality of the included studies was generally good. However, according to the COSMIN guidelines, the measurement properties were rated as poor to fair. Notably, except for the Chinese versions, the cross-cultural adaptations of the scale explicitly stated that “no changes were made to the content structure or criteria; therefore, only reliability was verified.” Consequently, content validity—which is a crucial measurement property—was inadequately reported (22). Content validity should be rigorously evaluated by assessing the relevance and comprehensiveness of the items (27), with particular attention paid to the target population’s perceptions of the scale. For instance, Zhi et al. (28) employed cognitive interviews with the target population during the cross-cultural adaptation of a trauma-informed care attitude scale, which improved participants’ understanding and acceptance of the items and minimized measurement errors stemming from misinterpretation. Furthermore, the analysis of construct validity was insufficient, reported in only six of the 14 included studies. Construct validity reflects the degree to which a scale measures the specific theoretical trait or structure it purports to measure, representing the alignment between empirical data and underlying theory. Therefore, future cross-cultural adaptations must prioritize the robust evaluation of construct validity. Regarding internal consistency, all included studies reported appropriate psychometric properties, with Cronbach’s *α* coefficients ranging from 0.85 to 0.965, indicating high reliability across different cultural contexts.

### 5.2 Reliability analyses and factor structure

None of the included studies explicitly reported the theoretical model underlying the scale. The selection of an appropriate factor analysis method is contingent upon whether the scale is grounded in a theoretical framework, and the lack of such justification led to suboptimal methodological choices. The COSMIN guidelines recommend the use of CFA over EFA when the scale structure is hypothesized *a priori* based on theoretical models. CFA allows for a more detailed examination of the relationships between items and latent factors, as well as the direct or indirect associations among factors (29). For example, Chen et al. (30, 31) appropriately utilized both EFA and CFA, demonstrating good model fit indices. However, with the exception of the Chinese versions, no other cross-cultural adaptations reported the application of factor analysis (neither EFA nor CFA). Consequently, these studies received a “poor” quality rating for construct validity and an “insufficient” (−) rating for overall psychometric properties.

### 5.3 Criterion validity and hypothesis testing

This review identified a widespread methodological issue regarding the inappropriate establishment of a “gold standard” for criterion validity, which was frequently conflated with hypothesis testing. According to the COSMIN guidelines, patient-reported outcome measures generally lack a true gold standard, unless a newly developed abbreviated scale is being validated against the original, full-length version. In practice, researchers often erroneously utilize well-established scales as the gold standard for newly developed instruments; methodologically, this approach falls under the domain of hypothesis testing rather than criterion validity (14). Among the five studies that reported criterion validity, Chen et al. (30, 32) utilized the St. Mary’s Hospital Sleep Questionnaire (SMH) and He et al. (33) used the Pittsburgh Sleep Quality Index (PSQI) as the gold standard, none of which met the COSMIN criteria. For objective sleep indicators, PSG is recognized as the definitive gold standard. Murata et al. (34) and Rollo et al. (35) appropriately adopted PSG to test criterion validity; however, the correlation coefficient reported by Murata et al. (34) failed to reach the threshold of 0.7, resulting in a methodological quality rating of merely “good” rather than “very good.”

### 5.4 Cross-cultural validity and measurement error

None of the 14 included studies assessed cross-cultural validity, and only one study reported on measurement error. Cross-cultural validity is typically evaluated through measurement invariance testing or differential item functioning (DIF) analysis to ensure that scale items perform consistently across diverse cultural groups (36). Furthermore, measurement error encompasses both systematic and random errors. A fundamental principle is that if the Minimal Detectable Change (MDC) exceeds the Standard Error of Measurement (SEM), the observed change in the scale score is likely attributable to random error rather than a true systematic change. For quantitative data, the SEM can be derived from test–retest reliability, whereas for qualitative data, percentage agreement can be calculated (36). We strongly recommend that future research adhere to the COSMIN checklist to comprehensively evaluate the aforementioned measurement properties of the RCSQ. Specifically, future studies should concurrently assess inter-rater and intra-rater reliability and calculate measurement error to enhance the methodological rigor and scientific validity of the instrument.

A total of six of the 14 included studies (42.9%) were conducted in China, and most of the remaining studies were from other Asian countries (Thailand, Japan, and South Korea), resulting in a clear geographic bias toward Asian populations. None of the included studies assessed the cross-cultural validity or measurement invariance of the revised RCSQ, which is a critical deficiency. The psychometric properties of the Chinese version of the RCSQ are well-documented, with relatively large sample sizes, but it is unclear whether these properties can be generalized to other cultural and linguistic contexts—this is because sleep assessment is influenced by cultural perceptions (e.g., different cultural understandings of “sleep quality”) and linguistic expression (e.g., translation differences may affect item comprehension). The lack of cross-cultural validity data means that the revised RCSQ cannot be directly applied to non-Asian populations without further validation, and the generalizability of the present study’s findings is limited to Asian ICU and hospitalized patients. Future studies should conduct cross-cultural validation of the RCSQ across different continents and cultural backgrounds and test the measurement invariance of the scale to improve its cross-cultural applicability.

### 5.5 Heterogeneous populations and generalizability of validity evidence

The included studies covered highly heterogeneous populations, including ICU patients, older inpatients, orthopedic perioperative patients, and acute stroke patients. Sleep patterns, perception of sleep quality, and clinical disruptions differ markedly across these groups. The revised RCSQ was initially designed for ICU patients, but direct validation and application to other populations remain insufficient. This heterogeneity limits the stability of the validity evidence and weakens the generalizability of the findings to non-ICU populations. Future studies should conduct subgroup analyses or targeted validation for specific populations to improve the applicability and interpretability of the scale.

### 5.6 Clinical implications and research directions

The GRADE evidence quality ratings provide critical references for the clinical application and subsequent research of the revised RCSQ. The overall “moderate-to-high” level of evidence confirms robust support for its core psychometric properties (e.g., internal consistency and structural validity). High-quality evidence is mainly concentrated in the Chinese, Thai, and Brazilian Portuguese versions, which are characterized by adequate sample sizes and comprehensive validation of measurement properties. These versions can be prioritized for sleep assessment and the evaluation of intervention effectiveness in populations such as ICU and orthopedic perioperative patients. In contrast, moderate-quality evidence, largely due to smaller sample sizes or incomplete validation of measurement properties, requires application to be combined with objective sleep indicators (e.g., PSG) to reduce uncertainty. In particular, the minor language versions (e.g., Japanese and Korean) need to enhance evidence quality by expanding sample sizes and completing the assessment of all validation domains. Notably, all included studies performed well in terms of risk of bias and result consistency, with differences observed only in imprecision. This indicates strong cross-linguistic and cross-population stability of the revised RCSQ. However, future efforts should focus on addressing the evidence gaps in minor language versions and supplementing research on cross-cultural validity, thereby further strengthening the scale’s global application value.

The revised RCSQ has good internal consistency (Cronbach’s *α* 0.850–0.965) in ICU patients, and its simple structure and short completion time (1–10 min) make it a practical subjective sleep assessment tool for ICU clinical practice. However, due to insufficient evidence of structural validity and criterion validity, it can be used for rapid screening of sleep quality in ICU patients but not for accurate quantitative evaluation of sleep structure. The results should be combined with clinical observations and other objective indicators (e.g., actigraphy) for a comprehensive assessment. For older inpatients, perioperative orthopedic patients, and acute stroke patients, the revised RCSQ also shows good internal consistency, but the current research sample size is small and the evidence is insufficient. It can be used as a preliminary sleep assessment tool in these populations, but further validation studies with large samples are needed before widespread clinical application; the cutoff value of the scale should be adjusted according to the characteristics of different populations.

## 6 Strengths and limitations of this study

A key strength of this study lies in the rigorous and appropriate methodological framework adopted, which is essential for minimizing potential bias. This study is the most recent systematic review focusing on the psychometric properties of the Richards-Campbell Sleep Questionnaire (RCSQ) across different countries. By adhering to such a standardized and internationally recognized evaluation tool, the study enhances the reliability and validity of its findings, ensuring that the assessment of the scale’s properties is conducted in a consistent and rigorous manner. Another notable strength is the implementation of a validated, highly sensitive search strategy to identify relevant studies, which maximizes the comprehensiveness of literature retrieval.

However, this review has several limitations. First, only Chinese- and English-language studies were included, introducing language bias. We only included studies with primary psychometric evaluations; clinical trials with secondary data were excluded (consistent with the aim but may reduce yield). Only 14 studies were included, and most versions were based on small sample sizes. High patient heterogeneity (e.g., ICU, older, orthopedic, and stroke patients) limits unified conclusions. Key properties (content validity, cross-cultural validity, and measurement error) were underreported. In the initial draft of this manuscript, non-standard labels (e.g., ‘vague’ and ‘bad’) were used for the COSMIN risk of bias assessment. In this revision, all non-standard terms have been fully revised to the official COSMIN terminology (e.g., ‘doubtful’ and ‘inadequate’), and all tables (Tables 3, 4) and the main text have been reviewed and corrected to ensure consistency with the 2018 COSMIN guideline. This study only used narrative synthesis to analyze the psychometric properties of the revised RCSQ without meta-analysis, which may lead to the inability to quantitatively synthesize the effect size of each measurement property. The results are more descriptive and lack quantitative support to a certain extent.

## 7 Conclusion

The revised Richards-Campbell Sleep Questionnaire demonstrates good and stable internal consistency across all included versions. However, supporting evidence for content validity, structural validity, criterion validity, cross-cultural validity, measurement error, and responsiveness remains limited or inconclusive. Future cross-cultural adaptation and validation studies should strictly follow the updated COSMIN guidelines, including a full RoB assessment, quality criteria, and GRADE-adapted certainty of evidence.

## Data Availability

All data generated or analysed during this study are included in this published article. Or the raw data supporting the conclusions of this article will be made available by the corresponding authors or the first author, without undue reservation.
