# Supplementary material for: Assessing psychometric properties of the revised Richards-Campbell Sleep Questionnaire based on COSMIN: a systematic review
Source: Front Med (Lausanne). 2026 May 7;13:1826844. doi: 10.3389/fmed.2026.1826844 (PMC13191367; doi:10.3389/fmed.2026.1826844)
Supplement: Supplementary file 1 [file Data_Sheet_1.docx]

**Each database search strategy**

**WanFang: 6**

Title or keywords: ("Richards-Campbell" or "RCSQ") and ("questionnaire" or "scale" or "instrument") and ("reliability" or "validity" or "cross-cultural" or "measurement properties" or "measurement characteristics")

**China National Knowledge Network (CNKI) : 6**

SU = (' Campbell Richards' + 'RCSQ') * (' questionnaire '+' scale '+' tools') * (' reliability '+' validity '+' cross-cultural '+' geodesy characteristics' + 'measurement attributes)

**VIP (VIP) : 6**

U=(Richards-Campbell OR RCSQ) AND (Questionnaire OR scale OR tool) AND (reliability OR validity OR cross-cultural OR measurement properties OR measurement attributes)

**China Biomedical Literature Service System-SinOMED: 6**

1 "Richards-Campbell "[Common field: Intelligence] OR "RCSQ"[Common field: Intelligence]

2 "Questionnaire "[Common field: Intelligence] OR" Scale "[Common field: intelligence] OR "Tool "[Common field: intelligence]

3 "Reliability "[common field: intelligence] OR" validity "[Common field: intelligence] OR "cross-cultural "[Common field: intelligence] OR" measurement characteristics "[Common field: intelligence] OR "measurement attributes "[Common field: intelligence]

4 1 AND 2 AND 3

**PubMed：80**

#1 Richards-Campbell Sleep Questionnaire

#2 RCSQ

#3 #1 OR #2

#4 (instrumentation[sh] OR methods[sh] OR Validation Studies[pt] OR Comparative Study[pt] OR “psychometrics”[MeSH] OR psychometr*[tiab] OR clinimetr*[tw] OR clinometr*[tw] OR “outcome assessment (health care)”[MeSH] OR outcome assessment[tiab] OR outcome measure*[tw] OR “observer variation”[MeSH] OR observer variation[tiab] OR “Health Status Indicators”[Mesh] OR “reproducibility of results”[MeSH] OR reproducib*[tiab] OR “discriminant analysis”[MeSH] OR reliab*[tiab] OR unreliab*[tiab] OR valid*[tiab] OR coefficient[tiab] OR homogeneity[tiab] OR homogeneous[tiab] OR “internal consistency”[tiab] OR (cronbach*[tiab] AND (alpha[tiab] OR alphas[tiab])) OR (item[tiab] AND (correlation*[tiab] OR selection*[tiab] OR reduction*[tiab])) OR agreement[tiab] OR precision[tiab] OR imprecision[tiab] OR “precise values”[tiab] OR test–retest[tiab] OR (test[tiab] AND retest[tiab]) OR (reliab*[tiab] AND (test[tiab] OR retest[tiab])) OR stability[tiab] OR interrater[tiab] OR inter-rater[tiab] OR intrarater[tiab] OR intra-rater[tiab] OR intertester[tiab] OR inter-tester[tiab] OR intratester[tiab] OR intra-tester[tiab] OR interobserver[tiab] OR inter-observer[tiab] OR intraobserver[tiab] OR intra-observer[tiab] OR intertechnician[tiab] OR inter-technician[tiab] OR intratechnician[tiab] OR intra-technician[tiab] OR interexaminer[tiab] OR inter-examiner[tiab] OR intraexaminer[tiab] OR intra-examiner[tiab] OR interassay[tiab] OR inter-assay[tiab] OR intraassay[tiab] OR intra-assay[tiab] OR interindividual[tiab] OR inter-individual[tiab] OR intraindividual[tiab] OR intra-individual[tiab] OR interparticipant[tiab] OR inter-participant[tiab] OR intraparticipant[tiab] OR intra-participant[tiab] OR kappa[tiab] OR kappa’s[tiab] OR kappas[tiab] OR repeatab*[tiab] OR ((replicab*[tiab] OR repeated[tiab]) AND (measure[tiab] OR measures[tiab] OR findings[tiab] OR result[tiab] OR results[tiab] OR test[tiab] OR tests[tiab])) OR generaliza*[tiab] OR generalisa*[tiab] OR concordance[tiab] OR (intraclass[tiab] AND correlation*[tiab]) OR discriminative[tiab] OR “known group”[tiab] OR factor analysis[tiab] OR factor analyses[tiab] OR dimension*[tiab] OR subscale*[tiab] OR (multitrait[tiab] AND scaling[tiab] AND (analysis[tiab] OR analyses[tiab])) OR item discriminant[tiab] OR interscale correlation*[tiab] OR error[tiab] OR errors[tiab] OR “individual variability”[tiab] OR (variability[tiab] AND (analysis[tiab] OR values[tiab])) OR (uncertainty[tiab] AND (measurement[tiab] OR measuring[tiab])) OR “standard error of measurement”[tiab] OR sensitiv*[tiab] OR responsive*[tiab] OR ((minimal[tiab] OR minimally[tiab] OR clinical[tiab] OR clinically[tiab]) AND (important[tiab] OR significant[tiab] OR detectable[tiab]) AND (change[tiab] OR difference[tiab])) OR (small*[tiab] AND (real[tiab] OR detectable[tiab]) AND (change[tiab] OR difference[tiab])) OR meaningful change[tiab] OR “ceiling effect”[tiab] OR “floor effect”[tiab] OR “Item response model”[tiab] OR IRT[tiab] OR Rasch[tiab] OR “Differential item functioning”[tiab] OR DIF[tiab] OR “computer adaptive testing”[tiab] OR “item bank”[tiab] OR “cross-cultural equivalence”[tiab])

#5 #3 AND #4

**Embase：17**

#1 Richards-Campbell Sleep Questionnaire:ti,ab,kw

#2 RCSQ:ti,ab,kw

#3 #1 OR #2

#4 (psychometr* OR clinimetr*):ti,ab,kw

#5 (valid* OR reliab* OR unreliab*):ti,ab,kw

#6 (internal consistency):ti,ab,kw

#7 (cronbach* AND (alpha OR alphas)):ti,ab,kw

#8 (test-retest OR stability OR repeatab* OR reproducib*):ti,ab,kw

#9 (interrater OR inter-rater OR intrarater OR intra-rater OR interobserver OR inter-observer OR intraobserver OR intra-observer):ti,ab,kw

#10 (intraclass AND correlation*):ti,ab,kw

#11 (factor analysis OR factor analyses OR dimension* OR subscale*):ti,ab,kw

#12 (observer variation OR coefficient of variation OR precision OR imprecision):ti,ab,kw

#13 (ceiling effect OR floor effect):ti,ab,kw

#14 (responsiv* OR sensitiv* OR minimal detectable change):ti,ab,kw

#15 (measurement error OR standard error of measurement OR variability OR uncertainty):ti,ab,kw

#16 (cross-cultural OR measurement invariance OR differential item functioning OR DIF):ti,ab,kw

#17 (validation study OR discriminant analysis OR hypothesis testing):ti,ab,kw

#18 (kappa OR agreement OR concordance):ti,ab,kw

#19 #4 OR #5 OR #6 OR #7 OR #8 OR #9 OR #10 OR #11 OR #12 OR #13 OR #14 OR #15 OR #16 OR #17 OR #18

#20 #3 AND #19

**CINAL：26**

S1

Richards-Campbell Sleep Questionnaire OR RCSQ

S2

internal consistency OR ceiling effect OR validity OR coefficient of variation OR observer variation OR psychometric OR validation study OR discriminant analysis OR precision

S3

(S1 AND S2)
